# The suppressive role of nanoencapsulated chia oil against DMBA-induced breast cancer through oxidative stress repression and tumor genes expression modulation in rats

**DOI:** 10.1007/s11033-022-07885-1

**Published:** 2022-09-05

**Authors:** Aida I. El makawy, Dalia M. Mabrouk, Shaimaa E. Mohammed, Sekena H. Abdel-Aziem, Heba A. Abd EL-Kader, Hafiza A. Sharaf, Dalia A. Youssef, Faten M. Ibrahim

**Affiliations:** 1 Cell Biology Department, Biotechnology Research Institute, National Research Centre, Giza, P.O.12622, Egypt; 2 Nutrition and Food Sciences Department, Food Industries and Nutrition Research Institute, National Research Centre, Giza, P.O.12622, Egypt; 3 Pathology Department, Medical Research and Clinical Studies Institute, National Research Centre, Giza, P.O.12622, Egypt; 4 Pests and Plant Protection Department, Agricultural and Biology Research Institute, National Research Centre, Giza, P.O.12622, Egypt; 5 Medicinal and Aromatic Plants Research Department, Pharmaceutical and Drug Industries Research Institute, National Research Centre, Giza, P.O.12622, Egypt

**Keywords:** Fatty acids, Nanotechnology, Oil encapsulation, Breast cancer, Tumor suppressor genes, Oxidative stress, Tissue architecture

## Abstract

**Background:**

Chia oil is high in omega-3 fatty acids, which have been linked to a lower risk of many diseases, including cancer. Oil encapsulation is a method that holds promise for maintaining oil content while enhancing solubility and stability. The purpose of this study is to prepare nanoencapsulated Chia oil and investigate its suppressive effects on rat chemically induced breast cancer.

**Methods:**

The oil was extracted from commercial Chia seeds and their fatty acids were analyzed using Gas Chromatography–mass spectrometry (GC/MS). Sodium alginate was used as a loading agent to create the Chia oil nanocapsules. The DPPH assay was used to assess the oil nanocapsules' capacity to scavenge free radicals. Breast cancer induction was done by single dose subcutaneously administration of 80 mg/kg dimethylbenz (a) anthracene (DMBA). Models of breast cancer were given Chia oil nanocapsules orally for one month at doses of 100 and 200 mg/kg. Through measuring intracellular reactive oxygen species (ROS) and protein carbonyl, assessing the gene expression of tumor suppressor genes (BRCA 1 & 2, TP53), and conducting histopathological analysis, the suppressive effect of Chia oil nanocapsules was examined.

**Results:**

The increase in ROS and PC levels brought on by DMBA was significantly decreased by the administration of Chia oil nanocapsules. In tumor tissue from rats given Chia oil nanocapsules, the mRNA expression levels of BRCA1, BRCA2, and TP53 were controlled Histopathological analysis clarified that the tissue architecture of breast tumors was improved by nanocapsules management.

**Conclusions:**

These findings demonstrate the ability of Chia oil nanocapsules to inhibit cancer cells in the rat breast.

## Background

Breast cancer is a leading cause of death among women and is one of the mortality and ill health causes [1]. It is the most common cancer among women in both developed and developing nations is breast cancer. The highest incidence rates are found in high-income countries, whereas the highest mortality rates are found in low-income states. Factually, it is estimated that 1.7 million new cases of breast cancer will be diagnosed in the developing world over the next few years, and the huge disparity in mortality rates will persist, with the developing world accounting for 70% of breast cancer deaths [2]. The researchers were driven to look for new and potent medications with fewer side effects because of the rising death rate as well as the unfavorable effects of anticancer medications [3].

Animal models of chemically induced carcinogenesis are trustworthy and frequently employed to assess the therapeutic/diagnostic potential of drugs in cancer research. Chemical carcinogens called polycyclic aromatic hydrocarbons (PAH), like 7,12-dimethylbenz (a) anthracene (DMBA), are frequently used to induce mammary carcinogenesis in rats [4]. Carcinogenesis comprises disruption and interruption of tissue redox balance, producing oxidative stress that caused cellular damage through lipid peroxidation and finally results in cellular and subcellular changes [5].

Fats and oils are the most important energy sources as well as carriers of various nutrients and fat-soluble vitamins. Lipids are one of the main structural materials of all cells and organs in the body and serve a variety of biological functions. Essential fatty acids, vitamins A, D, E, and K, and other nutrients found in fats are crucial for human health [6]. Essential oils are well known for their ability to target cancerous cells and can increase the effectiveness of chemotherapy medications. Its mechanism is based on the stimulation of a mitotic arrest through the targeting tubulin, which leads to the activation of the mitotic checkpoint and apoptosis [7]. People are now more aware than ever before of the connection between food and health [8]. It is well known that polyunsaturated fatty acids (PUFAs) have health-improving qualities [9]. Since the human body is unable to synthesize omega-3 and omega-6 essential fatty acids (EFAs), they must be consumed through diet [8]. Chia seed is a potential source of omega-3 PUFAs [10]. *Salvia hispanica *L., also known as chia seeds, is a member of the Lamiaceae family and is a high-quality source of plant oils [11]. Chia oil contains a high amount of omega-3 fatty acids, in particular a-linolenic acid, and 20% omega-6 fatty acids, especially linoleic acid [12]. Chia's high content of omega-3 is allied with the risk of reduction of coronary heart diseases, being antithrombotic, anti-inflammatory, hypertension, diabetes, rheumatoid arthritis, autoimmune diseases, and cancer [13].

Edible oils’ original characteristics are altered by nanotechnology, improving their quality, safety, and bioavailability [14]. For encapsulating, preserving, and creating nanocarriers for biologically active compounds, oil-based nanoemulsions are particularly intriguing. The surfactant’s high bioavailability and gravitationally stability are what give nanoemulsion-based delivery systems their properties [15]. Aside from materials unrelated to food, alginate is the best encapsulating substance because of its compatibility and safety. Alginates are frequently used for emulsification in the forms of calcium or sodium alginate [16]. Chia oil encapsulation is a potentially effective way to keep the oil content while enhancing its solubility and stability [17].

Research is increasingly showing the serious roles of oxidative stress and inflammation in the development of cancer. Inflammatory responses have been shown by Greten and Grivennikov to be important mechanisms of tumorigenesis and cancer promotion [18]. In the pathophysiological mechanisms of cancer, disruption in the expression of tumor suppressor and apoptotic genes is a critical factor [19]. In addition, several inflammatory, oxidative stress dysregulated pathways are involved in the initiation and development of cancer [20].

Accordingly, our goal in this study was to investigate the feasibility of using Chia oil in the formulation of nanocapsules using sodium alginate as a loading material. Also, look into the suppressor role of Chia oil nanocapsules against chemically induced breast cancer through oxidative stress and gene expression.

## Materials and methods

### Chemicals

Sodium alginate [Manutex FAV, ISP Alginates, (UK)], calcium chloride (Merck, Germany), and Tween 20, (Merck KGaA, Darmstadt, Germany). The 7,12-dimethylbenz(a)anthracene (DMBA), was purchased from Sigma chemical company (St. Louis, MO, USA). The seeds of Chia were purchased from Harraz - Agricultural Seeds, Spices & Medical Plants Co. Egypt and were characterized in the Egyptian agricultural museum.

### Preparation of chia oil

Oil was extracted from chia seeds powdered (500) g on cold by soaking with petroleum ether 40–60. The extraction continued until exhaustion and the petroleum ether extract was evaporated under a vacuum at 35 °C using a rotary evaporator until complete solvent removal. The dried solvent-free extract was used to prepare saponifiable fractions which were studied by GC/MS analysis.

#### Saponification of petroleum ether extract

According to Tsuda et al. [21], 0.5 gm of the petroleum ether extract residue was refluxed for 6 h with 0.5 N alcoholic KOH (100 ml) in a boiling water bath. The saponified extract was concentrated to about a third of its original volume. The cooled reaction mixture was diluted with equal volumes of distilled water and extracted thoroughly with ether (negative test of sterols). The combined ethereal extract was washed with water several times until it was alkalinity-free before being dehydrated over anhydrous sodium sulfate. The residue was kept for GC/MS analysis after the ether was evaporated to dryness. The alkaline aqueous solution remaining after extraction of the unsaponifiable matter was acidified with hydrochloric acid to liberate the fatty acids, which were extracted several times with ether. The combined ethereal extract was washed several times with distilled water until acidity was removed, then filtered through anhydrous sodium sulfate and evaporated to dryness.

### Preparation of fatty acid methyl esters (FAME)

The residue of fatty acids obtained was dissolved in 50 ml absolute methanol, mixed with 0.25 ml sulphuric acid, refluxed for about 3 h, cooled, diluted with about 100 ml distilled water, and transferred to a separating funnel as the methodology of Finar [22]. The resulting fatty acid methyl esters were extracted several times with ether. The combined ethereal extract was washed several times with water until free from acidity and dehydrated over anhydrous sodium sulfate. The solvent was evaporated, and the residue was kept for GC/MS analysis.

### Preparation and investigation of nanocapsules using high-energy ultrasonic

In this study, emulsified nanocapsules were prepared using sodium alginate solution as an aqueous phase, Chia oil as the oil phase, according to Youssef and Abdelmegeed [23]. The alginate solution was procured by dissolving sodium alginate in deionized water. Then, 30 g of Chia oil containing emulsifier was added drop wising. A mechanical stirrer (Greave Mixer, England) was used to vigorously stir this mixture at room temperature until it was emulsified and appeared creamy. The emulsion thus formed was sonicated for 30 min using an ultrasonic cleaner set, model WUC-DO3H 290 W, and 60 Hz, and then sonicated for 3 min using a high energy ultra-sonication probe (model VCX750, 750 W, 20 kHz). Then, calcium chloride solution (cross-linking agent) was added briefly to the mixture and stirred, then sonicated, as mentioned previously. Finally, the phase separation of oil/ water Nano-emulsion occurred.

### Transmission electron microscopy (TEM)

The nanocapsule’s size and morphology were characterized by TEM. For this purpose, nanocapsules suspension was diluted with distilled water and deposited onto a carbon-coated copper grid, then examined by magnification × 20,000 and photographed.

### Nanocapsules scavenging activity

The free radical scavenging ability of oil nanocapsules was tested by DPPH radical scavenging assay, as described by Ibrahim et al. [24]. The ability of plant extractives to donate hydrogen atoms was determined by decolorizing a methanol solution of 2,2-diphenyl-1-picrylhydrazyl (DPPH). In methanol solution, DPPH produces a violet/purple color, which fades to shades of yellow in the presence of antioxidants. A 0.1 mM DPPH in methanol solution was made, and 2.4 ml of it was mixed with 1.6 ml of extract in methanol at various concentrations (12.5–150 g/ml). The reaction mixture was vortexed thoroughly and kept at room temperature for 30 min in the dark. At 517 nm, the mixture's absorbance was measured spectrophotometrically and BHT was used as a benchmark. The following equation was used to calculate the percentage of DPPH radical scavenging activity:

$$\% {\text{ DPPH percentage}}\, = \,\left[ {\left( {{\text{A}}0 - {\text{A1}}} \right)/{\text{A}}0} \right] \, \times { 1}00$$

A0 = the control absorbance, and A1 is the absorbance of the extracts/standard. The percentage of inhibition was plotted compared to concentration, and from the graph, the IC50 was calculated.

### Acute toxicity study 

The acute oral toxicity of Chia oil nanocapsules in Sprague–Dawley rats was estimated to evaluate any possible toxicity. Animals were tested by administering different doses by increasing or decreasing the dose, according to the response of animals, while the control group received normal saline according to OECD [25]. All groups were monitored for 48 h and daily for 14 days, or until there were early signs of toxicity and/or mortality. The LD50 was calculated to select the doses used in the biological experiment. The LD50 of chia oil nanocapsules was 2000 mg/kg therefore the selected doses were 1/20 and 1/10 of LD50 that equal 100 and 200 mg/kg bw.

### Anticancer biological activity evaluations

#### Animals

Adult female Sprague–Dawley of 160 ± 20 g (6–8 weeks) was obtained from the animal laboratory of our institution and was reserved for acclimatization for about 2 weeks. Animals were fed with a pellet diet and water ad libitum throughout the investigational time. Standard laboratory conditions were maintained under-regulated atmosphere (12:12 h light/dark cycles with an ambient temperature of 22 ± 3 ℃ and humidity at 50 ± 10. All animal experiments were carried out strictly following International Ethical guidelines and the National Institutes of Health Guide concerning the Care and Use of Laboratory Animals. The experiments were approved by the medical research ethics committee of the National Research Centre (Registration number 19164).

### Experimental design

Sixty-nine female Sprague–Dawley rats 6–8 weeks old, weighing 150 g, were used in this study. Firstly, 24 control rats were divided into four groups each containing 6 rats as follows: Group 1: Rats were orally gavaged with saline and used as negative control; Group 2: Rats were treated via gavages with corn oil as a vehicle Group 3: Rats were orally treated with Chia oil nanocapsules 100 mg/kg; Group 4: Rats were orally gavaged with 200 mg/kg Chia oil nanocapsules. Secondly, 45 female Sprague–Dawley rats were injected subcutaneously in the mammary region with a single dose of 80 mg/kg DMBA (Sigma-Aldrich; St. Louis, MO, USA) dissolve in 0.5 ml corn oil. After 4 months rat breast cancer models were divided into four groups: DMBA group animals remained without treatment; reference drug group in which animals were administrated with 5-fluorouracil 20 mg/kg/day; Chia oil nanocapsules 100 mg/kg group in which animals were orally gavaged with Chia oil nanocapsules 100 mg/kg/day; and Chia oil nanocapsules 200 mg/kg group wherein animals were orally gavaged with Chia oil nanocapsules 200 mg/kg/day. DMBA group contains 15 animals, while the others include 10 animals. The Nanoemulsions and reference drugs were administrated for 1 month.

### Tissue sampling

After completion of the experiment, blood samples were obtained from the inferior vena cava and collected in heparinized glass tubes, and were then centrifuged at 5000 rpm for 10 min. Plasma was separated and stored in aliquots at − 80 °C until analyzed. Then animals were sacrificed and mammary tumors and normal mammary glands of all test groups were quickly dissected and prepared for the different techniques.

### Estimation of intracellular reactive oxygen species (ROS) and protein damage

Reactive oxygen species levels were measured using reactive oxygen species Elisa kit for rat (Cat. No. SL1189Ra, Sunlong Biotech Co., Ltd). Protein carbonyl (PC) content was measured using the rat PC Elisa kit (SL 1055Ra, Sunlong Biotech Co., Ltd). Briefly, five wells were filled with serially diluted standards, so that the total volume in each well was 50 µl and the concentrations after dilution are 1500, 1000, 500, 225 and 125 pg/ml for ROS and 36, 24, 12, 6 and 3 ng/ml for PC. A well was kept as a control blank. Sample wells were filled with 40 ul sample dilution buffer followed by the addition of 10 µl sample (dilution factor 5). Wells were mixed with gentle shaking with consequent incubation for 30 min at 37 °C after being sealed with the closure plate membrane. The wells were washed using diluted washing buffer for five times. After discarding the washing buffer, 50 µl HRP-Conjugate reagent was added to each well except the control one. Once again incubation for 30 min at 37 °C followed by washing five times as mentioned previously. Chromogen solution A (50 µl) was added followed by Chromogen solution B (as a coloring step) to each well, wells were mixed with gentle shaking with consequent incubation for 15 min. at 37 °C. The reaction was terminated using 50 µl from stop solution and the wells color was changed from yellow to blue. Absorbance optical density was measured at 450 nm using the Microtiter Plate Reader. Known concentrations of standard and its corresponding reading OD is plotted on the log scale (x-axis) and the log scale (y-axis) respectively. The concentration in sample is determined by plotting the sample’s O.D. on the Y-axis. The original concentration is calculated by multiplying the dilution factor.

### Real-time quantitative PCR for gene expression

Total RNA was extracted from the tumor tissue using an Easy red total RNA extraction kit (Intronbio, Korea), according to manufacturer’s instructions. The concentration and purity of RNA was analyzed using NanoDrop™ 1000 Spectrophotometer (Thermo Fisher Scientific, USA). RNA (1 µg) was treated with RNase-free DNase kit (Thermo Fisher Scientific, USA) to remove any genomic DNA contamination and then cDNA was synthesized using RevertAid First Strand cDNA Synthesis Kit (Thermo Fisher Scientific, USA).. Three tumor suppressor genes; breast cancer gene 1 (BRCA1), breast cancer gene 2 (BRCA 2), and tumor suppressor gene (TP53) were tested in the study, and glyceraldehyde-3-phosphate dehydrogenase gene (GAPDH) was used as an internal control. Specific primers for studied genes BRCA1, F: TGA AGA CTG CTC GCA GAG TGA TA, R: AGC TTC CAG GTG AGC CAT TTC; BRCA2, F: TTGAGGACCCCAAGACCTGT, R: CCGGAGAGACAAA GGTGCA; TP53, F: GCA GAG TTG TTA GAA GGC, R: TTG AGA AGG GAC GGA AGA; GAPDH, F: AAC TTT GGC ATT GTG GAA GG, R: ACA CAT TGG GGG TAG GAA CA were purchased from willowfort.co.uk. RT-qPCR was performed in Rotor-gene Q Real-time PCR cycler (Qiagen) in a 20 µl mixture containing 10 µl, Master Mix (2×) (Thermo Fisher Scientific, USA), 1 μl of cDNA, 0.5 μl each of forward and reverse primers (10 pmol/μl), and nuclease-free water up to 20 μl. Gene expression data were normalized to GAPDH and analyzed using the 2^ − ΔΔ Ct^ method [26].

### Histopathological investigation

Samples of inguinal mammary glands were taken from all test groups and fixed in 10% neutral-buffered formalin for 72 h, dehydrated through graded alcohols, cleared using xylene and embedded in paraffin wax. Sections of 5 μm thickness were prepared using the microtome, stained with hematoxylin and eosin (Hx&E) for microscopic examination [27].

### Statistical analysis

Statistical analyses were done using Statistical Package for the Social Sciences (SPSS software version 16). The data were analyzed using a one-way analysis of variance (ANOVA) followed by Duncan’s test. The data expressed as mean ± SE and the probability (P) level less than 0.05 was considered to be statistically significant at P ≤ 0.05.

## Results

### Fatty acids profile

**Fig. 1:**
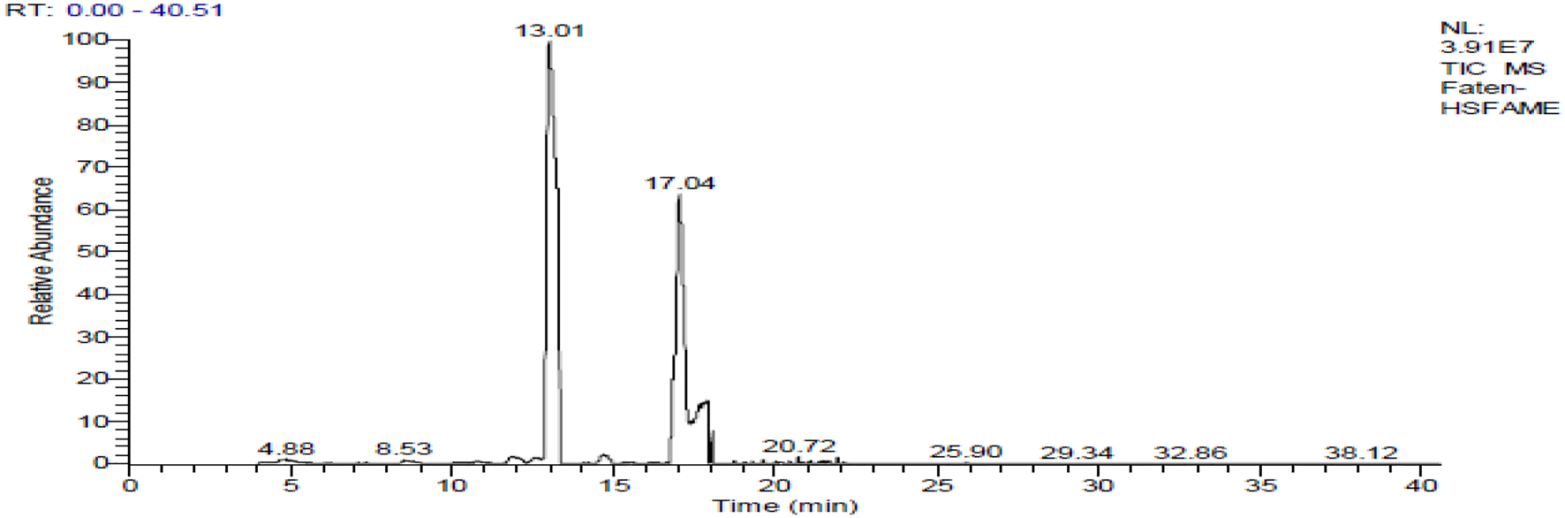
Chia oil methyl esters fatty acids chromatogram

The chromatogram of GC/MS analysis of the FAME of the Chia oil before encapsulation is represented in (Fig. 1). The results of GC/MS analysis of FAME of Chia oil were illustrated in Table 1, the Chia oil resulted in the identification of 17 compounds constituting 97.15% of the total peak area with Methyl hydroxypalmitate (39.16%), 6,9-Octadecdienoic acid methyl (linoleic acid; 27.40%) and 9,12,15-octadecatrienoic acid methyl ester (α-Linolenic acid; 11.96%) as major constituents. The unsaturated fatty acids constitute 41.55% of the total peak area.

**Table 1:** GC/MS analysis of fatty acids methyl esters of chia oil

No	R_t_	Compounds	M +	Molecular formula	Relative area %
1	8.54	12-Methyl tridecanoate	242	C_15_H_30_O_2_	0.41
2	10.68	9-Methyltetradecanoic acid methyl ester (Methyle myristate)	256	C_16_H_32_O_2_	0.05
3	10.75	Pentadecanoic acid methyl ester	256	C_16_H_32_O_2_	0.05
4	12.59	Hexadecadienoic acid methyl ester (Telfairic acid)	266	C_17_H_30_O_2_	0.36
5	12.71	9-Hexadecenoic acid methyl ester (Elaidic acid)	268	C_17_H_32_O_2_	0.34
6	12.97	2-Hydroxy pentadecanoic acid methyl ester. (Methyl hydroxypalmitate)	272	C_16_H_32_O_3_	39.16
7	13.29	Hexadecanoic acid,methyl ester (Methyl palmitate)	270	C_17_H_34_O_2_	12.46
8	14.68	14-Methyl hexadecanoic acid methyl ester	284	C_18_H_36_O_2_	0.98
9	14.75	Heptdecanoic acid methyl ester Margaric acid	284	C_18_H_36_O_2_	0.81
10	16.80	3,6-Octadecadienoic acid methyl ester	294	C_19_H_34_O_2_	1.43
11	17.04	6,9-Octadecdienoic acid methyl ester(6,9-Linoleic acid)	294	C_19_H_34_O_2_	27.40
12	17.41	9,12,15-Octadecatrienoic acid methyl ester(α-Linolenic acid, methyl ester)	292	C_19_H_32_O_2_	11.96
13	18.05	Octadecanoic acid methyl ester Methyl stearate	298	C_19_H_38_O_2_	1.59
14	20.99	6,9,12-Octaecatrienoic acid methyl ester ɣ-Linolenic acid methyl ester	292	C_19_H_32_O_2_	0.06
15	22.10	Eicosanoic acid methyl ester Arachidic acid	326	C_21_H_42_O_2_	0.05
16	23.37	Heneicosanoic acid methyl ester	340	C_22_H_44_O_2_	0.04
17	25.90	Docosanoic acid methyl ester	354	C_23_H_46_O_2_	0.04
	Total identified compounds				**97.19**

### Chia oil nanocapsules TEM characterization

**Fig. 2:**
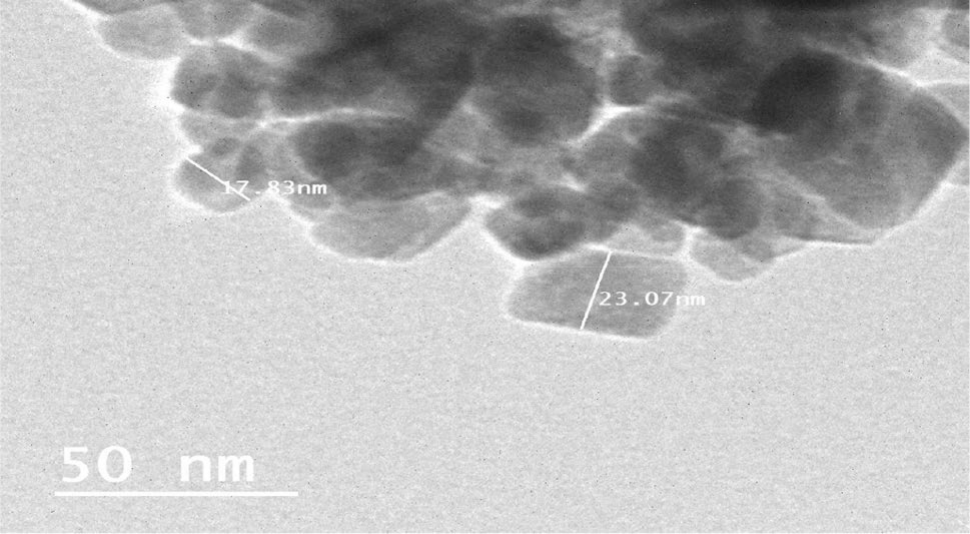
Transmission electron microscope (TEM) image of chia oil

To study the morphological shapes and size of prepared nano-formulations, nanocapsules were examined by TEM. The Chia oil nanocapsules were almost polygonal with a smooth surface as seen in (Fig. 2). The mean particle sizes were ranged between 10 and 35 nm.

### Chia oil nanocapsules DPPH radical scavenging activity

**Fig. 3:**
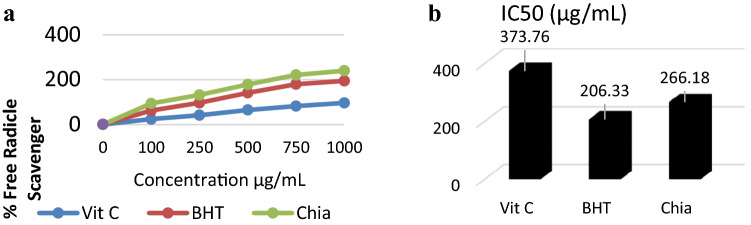
A DPPH radical scavenging activity and B IC50 of encapsulated chia oil. Data are expressed as mean ± SE (n = 6) for all tested dosages

Figure 3 shows the free radical scavenging activity and the IC50 of the Chia Nanocapsules and standards Vit C and BHT. The results showed that the Chia induced a concentration-dependent increase in the DPPH value. The IC50 of Chia oil was 266.18, while that of Vit C and BHT were 373.76, and 206.33 µg/ml, respectively. The free radical scavenging activity of Chia oil nanocapsules, Vit C and BHT, was in the following order Vit C > Chia oil > BHT.

### Effect of chia oil nanocapsules on oxidative stress markers in DMBA breast cancer rat model

Alterations in the number of analyzed biomarkers of oxidative stress in the blood serum of breast cancer model rats imply that the process of DMBA-carcinogenesis generates significant elevation (P ≤ 0.01) of oxidative stress. This is shown by an increase in reactive oxygen species (ROS) and protein carbonyl (PC) as seen in (Table 2). The administration of Chia oil nanocapsules at two doses diminished significantly (P ≤ 0.01) the elevation of ROS and PC levels than in DMBA-treated animals. Chia nanocapsules are more effective in reducing ROS and PC levels than 5-Flu.

**Table 2:** Effect of chia oil nanocapsules on oxidative stress markers levels.in DMBA breast cancer rat models.

Treatments	ROS	PC
Control	56.42 ± 1.68^e^	5.27 ± 0.15^e^
Corn oil	57.52 ± 1.55^e^	5.92 ± 0.20^e^
Chia oil nano 100 mg/kg	54.71 ± 1.74^e^	6.45 ± 0.41^e^
Chia oil nano 200 mg/kg	52.42 ± 1.52^e^	6.05 ± 0.31^e^
DMBA	650.42 ± 3.06^a^	16.17 ± 0.28^a^
DMBA ± 5-Flu	555.71 ± 4.37^b^	12.95 ± 0.26^b^
DMBA ± Chia oil nanocapsules 100 mg/kg	540.57 ± 3.55^c^	10.77 ± 0.11 cd
DMBA ± Chia oil nanocapsules 200 mg/kg	524.28 ± 7.85^d^	10.14 ± 0.16^d^

Data are expressed as mean ± SE (n = 6, P ≤ 0.05) for all tested dosages. Groups with unlike superscript letters were significantly (P ≤ 0.01) different.

### Role of chia oil nanocapsules on tumor suppressor genes expression

**Fig. 4:**
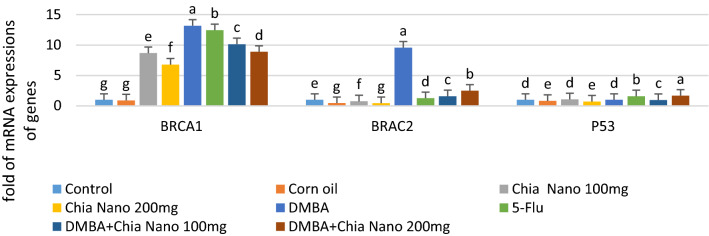
Effects of 5-Flurouracil and chia oil nanocapsules on BRCA1, BRCA2, TP53 expression in DMBA- breast tumor tissue of rats. Data are expressed as mean ± SE (n = 6, P ≤ 0.05) for all tested dosages. Groups with unlike superscript letters were significantly (P ≤ 0.01) different

As seen in (Fig. 4), the mRNA expression levels of BRCA1and BRCA2 were significantly up-regulated (13 and 12 fold, respectively) (P ≤ 0.05) in the DMBA group compared with the control group. In contrast, a significant decrease in the mRNA levels of BRCA1 and BRCA2 (P ≤ 0.05) was observed after chia nanocapsules (100, 200 mg/kg) treatment. A similar trend was observed in animals treated with 5-Flu. The mRNA expression levels of TP53 showed no obvious change between the DMBA treated group and the control group. Whereas, it revealed a significant upregulation (1.6 and 1.7 fold, respectively) (P ≤ 0.05) in tumor tissue treated with 5-flu and Chia oil nanocapsules (200 mg/kg) compared to the DMBA group. However, there was no significant difference in the breast tumor tissue treated with Chia nanocapsules (100 mg/kg).

### Histopathological investigation

**Fig. 5:**
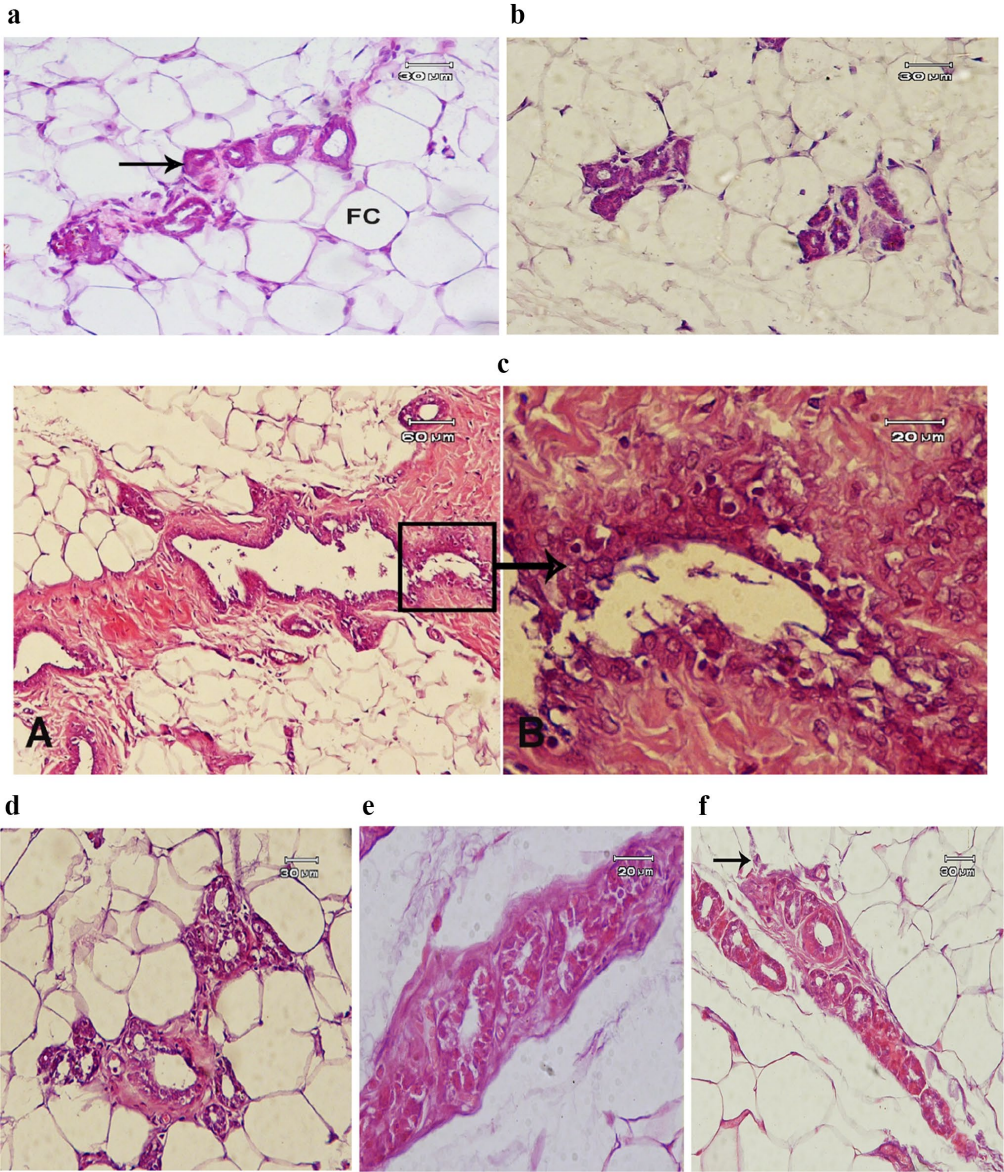
Section of mammary gland of: **a** control rat showing the normal picture of resting state with normal acini (arrow) and small ducts surrounded by connective tissue and adipose tissue (FC); **b** rat treated with Chia oil nanocapsules 200 mg/kg showing normal architecture of mammary gland; **c** rat treated with DMBA showing proliferation of ductal epithelial lining forming papillae and infiltrating the duct wall with desmoplasia of the breast stroma A. The higher magnification of tabulated part shows hyperchromasia and pleomorphism of the proliferating cells B; **d** DMBA-induced breast cancer treated with 5-Flu showing significant reduction of proliferation, no papillae formation, sings of desmoplasia still observed; **e** DMBA induced-breast cancer treated with Chia oil nanocapsules (100 mg/kg) showing signs of improvements represented in no papillae formation, no infiltration of duct wall and no desmoplasia, while, the proliferation of ductal epithelial cells is still present; **f** DMBA-breast cancer rat treated with Chia oil nanocapsules 200 mg/kg showing more improvement represented in reduction of proliferation of epithelial cells as well as desmoplasia (Hx&E)

Microscopic examination of the control mammary gland appeared that the gland was composed of tubular branching ducts and glandular alveoli; both lined by one to two layers of epithelium and had a well-defined lumen. The epithelium rests on a basement membrane were lined by a layer of myoepithelial cells and surrounded by connective tissue and adipose tissue (Fig. 5a). The breast tissue of normal female rats treated with low and high doses of Chia nanocapsules (Fig. 5b) showed more or less similar to those of the control group. In DMBA-induced breast tumor tissue (Fig. 5c) a proliferation of ductal epithelial lining forms papillae and infiltrates the duct wall with desmoplasia of the breast stroma (A) as well as, hyperchromasia and pleomorphism of the proliferating cells (B) were observed. However, the mammary gland of the rat breast cancer model that was treated with 5-fluorouracil showed a significant reduction of proliferation, no papillae formation, and signs of desmoplasia still observed (Fig. 5d). The breast cancer model treated with Chia oil nanocapsule 100 mg/kg revealed signs of improvements represented in absence of papillae formation, infiltration of duct wall, and desmoplasia, while the proliferation of ductal epithelial cells are still present (Fig. 5e). Although, the tissue of breast tumor rats treated with Chia nanocapsules at (200 mg/kg) showed more reduction in epithelial cells proliferation as well as desmoplasia (Fig. 5f).

## Discussion

Oxidative stress was stated to associate with the progression of numerous metabolic and chronic syndromes or cancers [28]. High ROS levels cause protein damage that comprises site-specific amino acid alteration, peptide chain destruction, and cross-linked reaction product aggregation [29]. Excessive amounts of free radicals can lead to cell damage and apoptosis, causing many diseases, such as cancer [30]. The DMBA breast cancer rat model produced more oxidative stress markers in this study, which is consistent with the study of Perillo et al. [31] who reported that reactive oxygen species (ROS) are a class of highly reactive molecules that have evolved as signaling pathway regulators. ROS elevation is contributed to a variety of pathologic conditions, including tumor promotion and progression. The toxic appearance of DMBA-induced oxidative stress was confirmed by Krishnamoorthy and Sankaran [32] who reported that DMBA-induced ROS is associated with a wide range of macromolecular damages in lipids, proteins, and nucleic acids. Our findings show that protein carbonization is higher in breast tumor tissue than in healthy tissue. This was in line with Aryal and Rao [33], who confirmed an increase in protein carbonization in breast cancer tissue, which earlier was attributed to higher levels of reactive oxygen species (ROS) and oxidative stress in tumor tissue [34].

Researchers are working on ways to prevent and treat breast cancer all over the world. Synthetic medicines develop as a result of rapid technological advancements, but due to their severe side effects, phytomedicine’s potential has received a lot of attention in recent decades [35]. The current study investigates the role of Chia oil encapsulation in breast cancer suppression. The findings showed that Chia nanocapsules could reduce the risk of breast cancer in rats. Several studies have confirmed Chia's anticancer properties. Mutar and Alsadooni [36] demonstrated that Chia seed (*Salvia Hispanica*) extract can be used to treat cancer due to its high protein content that making them a promising source of protein fractions with anticancer activity. Furthermore, Ahmed et al. [37] found that Chia seeds reduced the ROS levels significantly. This might be based on its high content of numerous antioxidants, including α-linolenic acid [12]. This complies with our results of Chia oil fatty acid analysis that proved its high content of α-linolenic acid. Armando and Compas [38] confirmed the antitumor properties of Chia seed oil attributed to the high content of alpha-linolenic acid (omega 3). Inline, alpha-linolenic acid has been discovered to have antitumor properties in a variety of cell lines, including breast, colon, and prostate cancer [39].

TP53 and BRCA are frequently implicated in the development of breast cancer [40]. The expression of TP53 was not changed in the DMBA tumor tissue. It is in line with the study of Rivlin et al. [41], in which there was no overexpression of TP53 in mammary tumor tissue generated by DMBA in mice. However, the treatment with 5-Flu and Chia oil nanocapsules, lead to its upregulation. TP53 activates the expression of pro-apoptotic genes, as well as inhibits the expression of anti-apoptotic genes, resulting in apoptosis induction [42]. The results suggest that the 5-Flu and Chia oil nanocapsules increase cancer cell apoptosis through a TP 53-dependent pathway. Analysis of the underlying molecular pathway reveals that Chia oil nanocapsules extract provokes apoptosis, which might be at least partially mediated through the upregulation of the tumor suppressor gene, TP53. Furthermore, an increase in the intensity of expression of this protein is associated with its enhanced concentration in the cell, which in turn is associated with greater damage occurring in the cell. According to Sznarkowska et al. [43], arrest of the cell cycle and the DNA damage repair require less active TP53 protein concentration, while apoptotic induction is necessary for it to have a greater level in the cell.

Our investigations revealed higher BRCA1 and BRCA 2-gene expression in DMBA-induced breast tissues. This could be due to the higher proliferation rate in malignant tissues which together with genetic instability may increase the need for more DNA damage repair. Similar results were observed by Wang et al. [44] who noticed upregulated mRNA levels of BRCA1 and BRCA2 in breast and ovarian cancer tissues. Chalabi et al. [48] clarified that over-expressed BRCA2 might play a role in the aggressiveness of breast tumors. BARCA1 & 2 expression in tumor tissue treated with Chia oil nanocapsules in two doses displayed significant downregulation. BRCA1 interacts with a variety of nuclear proteins, including BRCA2, and thus serves a variety of functions in the cell [45]. The amino terminal ring finger domain of BRCA1 is involved in estrogen receptor signaling repression, DNA repair modulation, and apoptosis. BRCA1’s carboxyl-terminal acidic domain acts as a transcriptional activator when linked to the DNA binding domain. Also, BRCA1 is involved in the control of cell cycle checkpoints and centromeres [46]. Satyananda et al. [47] speculated that BRCA2 high gene expression in breast cancers is associated with highly proliferative, higher-grade tumors. This is conspicuous in the histopathological observation of DMBA-breast cancer tissue, where the proliferation of ductal epithelial lining forming papillae shows hyperchromasia and pleomorphism of the proliferating cells. These effects were partially confirmed by Abou Zaid et al. [48], who found variation in the architecture of the DMBA mammary gland tissues, ranging from hyperplasia to anaplasia in the lining epithelium of the acini. Also, Ibrahim et al. [49] research discovered the capability of DMBA to increase the proliferation of rat breasts. Contrariwise, the study clarified that Chia oil encapsulation improved the tissue architecture of breast tumor animals. This proves the Chia nanocapsules’ ability to inhibit cancer cells in rat breasts. Numerous studies looked into the anti-proliferative properties of Chia seed oil in cancer cells [50]. Also, previous studies established the anticancer activity of Chia oil diets enriched with α-linoleic and their capability to thwart breast cancer by reducing the estrogen receptor a well-known promotor of breast cancer [51].

## Conclusion

Nanotechnology in micronutrients and its association with disease treatment is one of the domains of modern research needs. Nanotechnology is one of the key domains that can be used to prevent and slow the progression of certain diseases on a large scale. Based on current research, we can conclude that Chia oil nanocapsules are a promising adjuvant therapy for breast cancer. Chia oil encapsulation has shown positive effects, the most important of which are reduction of oxidative stress, modulation of tumor suppressor gene expression, and improvement of tissue architecture in breast tumor animals. All of these promising findings could pave the way for Chia nanocapsules to be developed as a chemopreventive drug to reduce the risk of breast cancer.

## Data Availability

The datasets supporting the conclusions of this article are included in this.

## Abbreviations

BRCA: Breast cancer gene
DMBA: Dimethylbenz (a) anthracene
DPPH: Diphenyl-1-picrylhydrazyl
FAME: Fatty acid methyl esters
GC/MS: Gas chromatography–mass spectrometry
ROS: Reactive oxygen species
TEM: Transmission electron microscopy
TP53: Tumor protein 53
PC: Protein carbonyl
